# Workshop on the Potential Role of Infectious Agents in Cardiovascular Disease and Atherosclerosis

**DOI:** 10.3201/eid0501.990131

**Published:** 1999


### Meeting Summaries

### Workshop on the Potential Role of Infectious Agents in Cardiovascular Disease and Atherosclerosis

Cardiovascular and cerebrovascular disease currently exact a substantial human and economic toll in the established market economies and are expected to become increasingly more prevalent in developing countries. The noncommunicable nature of coronary artery disease, myocardial infarction, stroke, and atherosclerotic plaques has now been questioned; *Chlamydia pneumoniae*, human cytomegalovirus, periodontal disease, *Helicobacter pylori*, and herpes simplex virus-1 have all been associated, to some extent, with these conditions. The strongest evidence links *C. pneumoniae*, then human cytomegalovirus, to coronary artery disease, but direct causation has not been established. Nevertheless, large antibiotic trials employing broad spectrum macrolides are now in progress, intended to treat *C. pneumoniae* infection in symptomatic cardiac atherosclerosis. In the future, if even a portion of vascular disease can be prevented with antimicrobial drugs, vaccines, and health education, the public health impact of these findings will be imposing.

To address this global public health issue, the National Center for Infectious Diseases and the National Center for Chronic Disease Prevention and Health Promotion, Centers for Disease Control and Prevention (CDC), Atlanta, Georgia, USA, cosponsored a multidisciplinary workshop of invited national and international researchers, clinicians, and state public health officials August 31–September 1, 1998. The workshop had two primary goals: 1) define the basic science and clinical applied research studies required to verify or refute the associations, within the context of other cardiovascular risk factors (emphasizing *C. pneumoniae* and human cytomegalovirus); 2) outline a preliminary public health strategy (noting the potential involvement of CDC) that will correctly address the role of infection in cardiovascular disease prevention and treatment.

Discussions of reviewed and new data reaffirmed that evidence of causality between any organism and atherothrombotic disease is sufficient neither to establish antibiotic treatment guidelines nor to generate a public health strategy that incorporates infection into the cardiovascular disease paradigm. To change the chronic course of disease, treatment must be specifically directed at the responsible agent and applied to the appropriate stage of pathogenesis in the population at risk. Thus, the pathobiologic interaction between infection, inflammation and free radicals, lipids, genetics, and other cardiovascular risk factors must be clarified with more sensitive, specific, and standardized tools. Reagants and methods must uniformly differentiate past exposure from active infection; they must examine how acute, recurrent, and persistent infection could initiate or aggravate atherosclerosis and acute arterial thrombotic events. Expansion of animal and in vitro models will add valuable information to human treatment trials and to epidemiologic studies that address the multifactorial nature of cardiovascular disease and focus on groups at risk. Laboratory innovations are needed to maximize interpretation of results and evaluate the impact of antimicrobial and non-antimicrobial interventions. Only in this way can a public health plan based upon sound scientific evidence be developed.

The following were among the recommendations of the workshop: 1) the basic epidemiology of *C. pneumoniae* should be defined, including the prevalence of past and active infection, risk factors for infection and the interaction of these determinants with traditional cardiovascular and cerebrovascular disease risk factors; 2) sensitive and specific diagnostic assays (both new and revised) for laboratory, animal, and clinical studies should be standardized to assess the true long- and short-term effects of infection and intervention; 3) pathobiologically relevant animal and in vitro models should be used to investigate the pathogenesis of single and multiple agents at different stages in the disease process, enhance the standardization of tools, and evaluate interventions; 4) activities should be linked to judicious antimicrobial use—examining baseline knowledge and use of antibiotics in cardiovascular disease, monitoring changes in disease incidence and prevalence with increasing antibiotic prescription, and investigating the effects of treatment on other microbes; 5) a close alliance should be formed between CDC, the National Institutes of Health, and investigators from diverse fields to advance these objectives in a timely fashion, accumulating high quality scientific evidence that will define the true role of infections and inflammation in atherosclerotic disease and the appropriateness of antimicrobial therapy.

For more information on the conference, contact Siobhán O'Connor, Centers for Disease Control and Prevention, 1600 Clifton Rd., NE, Mailstop C12, Atlanta, GA 30333, USA; tel: 404-639-1454; fax: 404-639-3039; e-mail: sbo5@cdc.gov.

### Workshop on Risks Associated with Transmissible Spongiform Encephalopathies (TSEs)

A workshop on the evaluation of risks posed by TSEs was held June 8-9, 1998, at the University of Maryland College Park, under the auspices of the Joint Institute for Food Safety and Applied Nutrition (JIFSAN), a cooperative venture of the University of Maryland and the U.S. Food and Drug Administration. The workshop, attended by 300 representatives from 17 countries, evolved in response to the need voiced by governmental agencies, industry groups, international organizations, and academic experts to evaluate TSE risks related to source materials, processing, and end products for animal and human use. Support for the workshop came from government agencies, industry groups, international organizations, and JIFSAN.

One goal of the workshop was to develop a framework of guiding principles for evaluating TSE risks. An initial draft outline of critical elements applicable to evaluations of TSE risk was presented. The next steps are to provide definitions for the critical elements and to develop an annotated outline that explains the relevance of each key element. A set of information tools (under development) to facilitate risk evaluation and access to TSE knowledge was demonstrated at the workshop.

The workshop 1) identified research needs and reviewed risks associated with sources of raw materials (material collection and procurement are primary control points for ensuring low risk of TSE transmission); 2) focused on inactivation schemes for TSE agents, which may be the most reliable way of reducing the level of TSE agent; 3) examined use of several categories of end products: foods, pharmaceuticals, cosmetics, blood and blood products, biologics, and tissues of animal and human origin; and 4) proposed strategies for TSE risk evaluation.

Representatives of governments, private organizations, and industry groups presented their approaches to the evaluation of TSE risks and found common themes: 1) zero risk does not exist; 2) decisions concerning public health issues must often be made in the absence of complete information; 3) risk analysis may be useful in understanding the key elements of a problem or situation; 4) the risk evaluation process must be responsive to rapidly changing information and new scientific data; 5) the risk evaluation process should be transparent, involving all partners (general public, regulated industry, government); 6) disagreements still exist concerning the appropriateness of models, assumptions, and methods; and 7) although both qualitative and quantitative approaches to TSE risk evaluation have merit, no single approach is applicable to all situations.

Workshop documents, including illustrated transcripts of the presentations, a draft outline of critical elements in TSE risk evaluations, observations on human and animal issues, and workshop overviews, are available at http://www.life.umd.edu/jifsan/tse.html).

### USDA Report on Potential for International Travelers to Transmit Foreign Animal Diseases to U.S. Livestock or Poultry

The U.S. Department of Agriculture has published a new report—The Potential for International Travelers to Transmit Foreign Animal Diseases to U.S. Livestock or Poultry—which explores the issue of animal disease transmission by human travelers. Millions of people travel internationally each year, for both pleasure and business, and thus may be at risk for being infected by animals and causing infections in animals.

The report highlights and summarizes what is currently known about the potential for human infection and human-to-animal transmission of foreign animal diseases. It focuses on the International Office of Epizootics List A diseases. These transmissible animal diseases, which may spread rapidly and have serious

Cardiovascular and cerebrovascular disease currently exact a substantial human and economic toll in the established market economies and are expected to become increasingly more prevalent in developing countries. The noncommunicable nature of coronary artery disease, myocardial infarction, stroke, and atherosclerotic plaques has now been questioned; *Chlamydia pneumoniae*, human cytomegalovirus, periodontal disease, *Helicobacter pylori*, and herpes simplex virus-1 have all been associated, to some extent, with these conditions. The strongest evidence links *C. pneumoniae*, then human cytomegalovirus, to coronary artery disease, but direct causation has not been established. Nevertheless, large antibiotic trials employing broad spectrum macrolides are now in progress, intended to treat *C. pneumoniae* infection in symptomatic cardiac atherosclerosis. In the future, if even a portion of vascular disease can be prevented with antimicrobial drugs, vaccines, and health education, the public health impact of these findings will be imposing.

To address this global public health issue, the National Center for Infectious Diseases and the National Center for Chronic Disease Prevention and Health Promotion, Centers for Disease Control and Prevention (CDC), Atlanta, Georgia, USA, cosponsored a multidisciplinary workshop of invited national and international researchers, clinicians, and state public health officials August 31—September 1, 1998. The workshop had two primary goals: 1) define the basic science and clinical applied research studies required to verify or refute the associations, within the context of other cardiovascular risk factors (emphasizing *C. pneumoniae* and human cytomegalovirus); 2) outline a preliminary public health strategy (noting the potential involvement of CDC) that will correctly address the role of infection in cardiovascular disease prevention and treatment.

Discussions of reviewed and new data reaffirmed that evidence of causality between any organism and atherothrombotic disease is sufficient neither to establish antibiotic treatment guidelines nor to generate a public health strategy that incorporates infection into the cardiovascular disease paradigm. To change the chronic course of disease, treatment must be specifically directed at the responsible agent and applied to the appropriate stage of pathogenesis in the population at risk. Thus, the pathobiologic interaction between infection, inflammation and free radicals, lipids, genetics, and other cardiovascular risk factors must be clarified with more sensitive, specific, and standardized tools. Reagants and methods must uniformly differentiate past exposure from active infection; they must examine how acute, recurrent, and persistent infection could initiate or aggravate atherosclerosis and acute arterial thrombotic events. Expansion of animal and in vitro models will add valuable information to human treatment trials and to epidemiologic studies that address the multifactorial nature of cardiovascular disease and focus on groups at risk. Laboratory innovations are needed to maximize interpretation of results and evaluate the impact of antimicrobial and non-antimicrobial interventions. Only in this way can a public health plan based upon sound scientific evidence be developed.

The following were among the recommendations of the workshop: 1) the basic epidemiology of *C. pneumoniae* should be defined, including the prevalence of past and active infection, risk factors for infection and the interaction of these determinants with traditional cardiovascular and cerebrovascular disease risk factors; 2) sensitive and specific diagnostic assays (both new and revised) for laboratory, animal, and clinical studies should be standardized to assess the true long- and short-term effects of infection and intervention; 3) pathobiologically relevant animal and in vitro models should be used to investigate the pathogenesis of single and multiple agents at different stages in the disease process, enhance the standardization of tools, and evaluate interventions; 4) activities should be linked to judicious antimicrobial useexamining baseline knowledge and use of antibiotics in cardiovascular disease, monitoring changes in disease incidence and prevalence with increasing antibiotic prescription, and investigating the effects of treatment on other microbes; 5) a close alliance should be formed between CDC, the National Institutes of Health, and investigators from diverse fields to advance these objectives in a timely fashion, accumulating high quality scientific evidence that will define the true role of infections and inflammation in atherosclerotic disease and the appropriateness of antimicrobial therapy.

For more information on the conference, contact Siobhán O'Connor, Centers for Disease Control and Prevention, 1600 Clifton Rd., NE, Mailstop C12, Atlanta, GA 30333, USA; tel: 404-639-1454; fax: 404-639-3039; e-mail: sbo5@cdc.gov.

